# Why does job insecurity influence employees proactivity differently? A dual-path model based on dualistic passion

**DOI:** 10.3389/fpsyg.2025.1697231

**Published:** 2025-12-12

**Authors:** Mingming Zhu, Kaiyuan He, Ziyang Qiang

**Affiliations:** 1 School of Business, Jiangsu Open University, Nanjing, China; 2 School of Economics and Management, Xiangnan University, Chenzhou, China; 3 School of Business, Hohai University, Nanjing, China

**Keywords:** job insecurity, proactive behavior, obsessive passion, harmonious passion, performance climate

## Abstract

**Introduction:**

Job insecurity (JI) has become a salient feature of contemporary work environments and a challenge that employees must address. However, scholars hold competing perspectives on the reasons why employees adopt different coping strategies in response to JI. To address this paradox, this study draws on Conservation of Resources (COR) theory and the Dualistic Model of Passion, to develop and test a dual-path model that explains how and why employees proactively respond to JI.

**Methods:**

We conducted a two-wave survey of 384 employees from China, examined the mediating roles of obsessive and harmonious passion and the moderating role of performance climate.

**Results:**

The results show that: (1) JI is only marginally and negatively associated with task proactivity; (2) JI positively predicts task proactivity through obsessive passion while negatively predicting it through harmonious passion; (3) Performance climate strengthens the positive indirect effect of JI on task proactivity via obsessive passion.

**Discussion:**

These findings clarify the competing mechanisms between JI and task proactivity, and extend COR theory by specifying the conditions under which resource investment occurs. This study provides practical guidance for organizations seeking to encourage employees to adopt adaptive responses while safeguarding employees’ sustainable engagement.

## 1 Introduction

Amid rapid industrial restructuring and a pervasive economic downturn, enterprises operate in an increasingly volatile and unpredictable competitive environment. This, in turn, poses a constant challenge to employees’ job stability. Undeniably, job insecurity (JI), which refers to individual perceived threat to the continuity and stability of their job, has become a salient feature of contemporary work environments for numerous employees (Ashford et al., 1989; Jiang et al., 2021). It is a challenge that employee must address. This is because JI is regarded as inherently stressful and undesirable (Peltokorpi and Allen, 2024; Shoss and Vancouver, 2024). A substantial body of research has characterized employees as passive victims who suffer from a lack of control over their future employment, and found that JI negatively affects a range of work-related and psychological outcomes (Keim et al., 2014; Jiang and Lavaysse, 2018).

Importantly, however, it would be a gross oversimplification to assume that employees are merely passive recipients of the negative consequences of JI (Shoss, 2017). From a practical perspective, employees experiencing JI often find themselves in a dilemma: to await passively for potential realization of job loss or to engage in proactive measures to mitigate it, even if such efforts may ultimately fail (Shoss and Vancouver, 2024). The prevailing scholarly consensus has focused on the adverse affective and cognitive outcomes of JI, emphasizing its role in triggering destructive coping strategies (Lee et al., 2018). However, there has been limited research on employees’ proactive agency. Although a number of recent studies suggest that JI may also lead to the activation of job preservation motivation (Shoss, 2017), most of the behavioral strategies discussed in the existing literature have limited constructive value, such as facades of conformity, political behavior, unethical behavior, or knowledge hiding (Huang et al., 2013; Hewlin et al., 2016; Shoss et al., 2022). These findings have led scholars to argue that it is essential to understand the individual-level psychological mechanisms through which employees proactively respond to JI, and to “consider how JI experiences interact with an individual’s broader sociopolitical context” (Klug et al., 2024).

In order to address this paradox, the present study draws on Conservation of Resources (COR) theory to explore the relationship between job insecurity and proactive behavior. This study focuses on proactive behavior as opposed to job performance for two primary reasons. First, job performance exhibits a time-lagged and cumulative characteristic influenced by a multitude of factors. This characteristic renders it less suitable for capturing employee’s divergent adaptive strategies in insecure work environments. Second, proactive behavior is a multi-motivationally driven behavioral pattern with different goals in response to complex work situations (such as enhancing competence, acquiring performance or providing instrumental self-representation; Strauss et al., 2017; Strauss and Parker, 2018; Guo et al., 2024).

According to COR theory, individuals are fundamentally motivated to gain and protect their valuable resources (Hobfoll et al., 2018). When faced with resource loss or threats, although individuals are inherently sensitive to the threat of resource loss, they also possess the capacity to protect and replenish their resource pools through active investment, rather than merely succumbing to helplessness (Hobfoll et al., 2018). Thus, when confronted with a resource threat like JI, COR theory provides a motivational explanation for why employees may be pushed to adopt opposing resource strategies, thereby generating different behavior. On the one hand, proactive behavior may serve as a critical resource investment strategy through which employees manage JI and regain control over their work environment. Indeed, “proactivity matters,” especially in uncertain and unpredictable contexts, because, as a resource investment strategy, it enables job-insecure employees not only to gain a sense of control over their environment and conserve critical resources (Cangiano et al., 2019; Parker et al., 2019), but also serves as an instrumental means for them to demonstrate their competence and value through self-representation (Strauss and Parker, 2018). On the other hand, owing to the resource-intensive nature, employees also may adopt resource-conservation strategies to reduce proactive behaviors when confronted with cognitive and emotional resource depletion caused by JI (He et al., 2022). This results in a state in which job-insecure employees are simultaneously subject to the forces of propelling and restraining. As a result, extant research has yielded disparate findings concerning the relationship between JI and proactive behavior (Griffin et al., 2007; Strauss and Parker, 2018).

To unravel the paradoxical relationship, the present study introduces the Dualistic Model of Passion (DMP) as a critical mechanism that operationalizes the resource investment and depletion pathways outlined by COR theory. DMP conceptualizes passion as comprising two distinct types: harmonious passion and obsessive passion, which arise from the different processes by which individuals internalize work-related activities into their identity (Vallerand et al., 2003). Organizational context, such as JI, plays a critical role in shaping these internalization processes (Vallerand et al., 2014). Thus, we propose that these two forms of passion can serve as the two motivational channels for the two core COR pathways. Specifically, by undermining employees’ basic psychological needs, a process that embodies resource depletion, JI may hinder employee autonomous internalization of work and thereby leading to resource conservation strategy (Van den Broeck et al., 2014; Griep et al., 2021). Conversely, by amplifying the salience of valued instrumental resources attached to the job (e.g., salary, identity), the threat of job loss in turn promotes more controlled internalization processes and thereby reinforcing obsessive passion (Klug et al., 2024). It captures the resource investment process in which individuals invest effort to prevent resource loss. Despite the established correlation between the dualistic passion and different adaptive outcomes, extant research frequently fails to consider the possibility that obsessive passion, a motivational structure, may also facilitate employee’s positive responses under adversity and threats (Vallerand et al., 2014). Therefore, to further explore the “black box” through which JI influences proactive behavior, this study integrates COR theory and DMP to explore the potential mediating mechanisms.

Building on COR theory, we further propose that the influence of JI on distinct forms of passion and, ultimately, proactive behavior, is likely moderated by the performance climate within the organization. COR theory suggests that resource evaluations are contingent upon contextual cues that signal the potential returns on resource investment (Hobfoll et al., 2018). Performance climate, which is characterized by the extant criteria of success and failure, social comparison with others, and intra-team competitive, provides such cues by explicitly linking effort with valued outcomes (Nerstad et al., 2013a). Under such conditions, employees experiencing JI may perceive proactive behavior as a worthwhile strategy for resource protection and gain, thereby channeling their energy into active responses. However, at the same time, such conditions may also lead to employees’ perception of higher instrumentality of work activities, which will undermine employee’s autonomous identification with the work. Accordingly, this study proposes that performance climate can function as a boundary condition in the relationship between JI, dualistic passion, and proactive behavior. By shaping employees’ cognitive evaluations of the instrumental value of proactive efforts, performance climate may influence how motivational mechanisms operate in the face of JI.

Our study contributes to the literature in several ways. First, by integrating COR theory with the DMP, we develop and test a dual-path model that explains the paradoxical mechanism through which JI influences employees’ proactive behavior. This model posits that JI can simultaneously promote proactivity through obsessive passion (resource investment mechanism) and inhibit it through harmonious passion (resource depletion mechanism), thereby clarifying the mixed findings in the literature. Second, this study challenges the prevailing notion of obsessive passion as exclusively detrimental, demonstrating its capacity to stimulate constructive behavioral tendencies under the distressing circumstance of JI. This contributes to a novel understanding of the DMP’s application in the realm of organizational adversity research. Third, by introducing performance climate as a boundary condition, we specify the contextual signals that reinforce the resource investment decisions, thereby extending COR theory.

## 2 Theory background and hypotheses development

### 2.1 Job insecurity and proactive behavior

Proactive behavior refers to individuals’ self-initiated, future-oriented action that aims to change and improve the situation or oneself (Parker et al., 2010). In essence, the purposes of such behavior entail an individual’s anticipation, preparation, provision of feedback and then eventually to “make things happen” (Parker et al., 2010; Strauss and Parker, 2018; Parker et al., 2019). Consequently, it is a resource-intensive process, entailing both benefits and risks (Strauss et al., 2017). A substantial body of research has emphasized that proactive behaviors are predominantly influenced by two factors: supportive environments and sufficient personal resources (Cangiano et al., 2019; Lebel et al., 2023). However, the competing perspective also suggests that proactivity can be a strategy for coping with adversity or dissatisfaction. For instance, Cai et al. (2019) argued that “*to be proactive, a certain level of dissatisfaction with the status quo is needed*.” From this perspective, JI, characterized by a threatening and uncertain *status quo*, may act as a stimulus for proactive behavior, which leads to mixed findings and theoretical debates about their relationship. COR theory provides an explanation for this competitive viewpoint.

On one hand, individuals are especially sensitive to resource loss, and even the threat of resource depletion can trigger significant stress and resource conservation tendencies (Hobfoll et al., 2018). In accordance with the resource depletion pathway, certain scholars have proposed a negative correlation between JI and proactive behavior. These researchers argue that the stress and anxiety generated by JI may deplete cognitive and self-regulatory resources, thereby engendering a defensive state in employees aimed at loss avoidance (Wang et al., 2015; Jiang, 2024). The phenomenon has been demonstrated to impair the capacity of employees to engage in future-focused activities such as planning and feedback seeking. For example, He et al. (2022)and Zhang J, et al. (2022) discovered that JI significantly reduces individuals’ capacity for self-regulation, thereby suppressing the enactment of proactive behaviors which require a substantial investment of resources.

On the other hand, however, these perspectives tend to overlook employee’s beliefs and motivations for proactively responding to threats. Researches based on job preservation motivation contend that employees are motivated to take initiative in preventing this loss because job loss has not yet occurred and work provides important social, economic, and psychological resources (Shoss, 2017). This aligns with the resource investment principle of COR theory, which posits that “*people must invest resources in order to protect against resource loss, recover from losses, and gain resources*” (Hobfoll et al., 2018). In other word, JI may activate individuals’ cognitive vigilance and forward-looking orientation, prompting them to address current challenges while also anticipating future opportunities. Accordingly, employees may respond to the threat of job loss by proactively engaging in resource investment action. For example, existing studies have found positive associations between JI and impression management motives (Huang et al., 2013; Ma et al., 2024), avoid-performance goal orientation (Long et al., 2022), and creativity (Teng et al., 2019). Van Hootegem et al. (2023) further demonstrated that JI enhances employee’ perceived need for development, which subsequently fosters their participation in developmental activities.

Based on this evidence, we argue that employees experiencing JI are likely to confront a dual motivational state: the propelling forces generated by retention or restoration of valuable work-related resources; the restraining forces generated by psychological threat and depletion. In other words, the relationship between JI and proactive behavior may reflect a suppressing effect, wherein competing psychological mechanisms operate simultaneously. Therefore, this study does not assume a direct relationship, and the following sections will draw on these two perspectives to systematically unpack the dual pathways.

### 2.2 Dual-path model based on DMP

#### 2.2.1 Job insecurity and passion

According to the DMP, work passion refers to employee’s strong inclination toward a self-defining work activity that individuals like, value, and are willing to invest time and resources (Vallerand et al., 2003). For passionate individuals, work activities are so vital as to become an integral part of their identity. It has been suggested that selection, valuation, and internalization are the prominent processes for the formation of passion (Vallerand et al., 2014). Depending on the degree to which the internalizing activity satisfies the basic psychological needs, individuals may internalize work in an autonomous to controlled manner (Ryan and Deci, 2003). Based on different internalization approaches, DMP differentiates two forms of passion: harmonious and obsessive, which reflect different ways of individual pursuing and internalizing valued work activities (Vallerand et al., 2003). The environment that employees face in the workplace directly influences how they internalize their work. Thus, JI, a salient environmental stressor, may shape how individuals evaluate and internalize their work activities, evoking distinct forms of work passion.

Obsessive passion results from a controlled internalization of work driven by intra- or interpersonal pressures, such as self-esteem, social acceptance, or instrumental work-related rewards (Vallerand et al., 2014). In other words, obsessive passion arises when individuals seek or maintain contingent self-esteem, which underscores the individual’s reliance on external validation and resources to sustain their self-worth (Astakhova, 2015). Employees with obsessive passion are therefore more likely to become compulsively involved in their valued work to secure or avoid the loss of personally important and valued resources, so as to obtain and maintain a positive self-evaluation (Lafrenière et al., 2011). Accordingly, although both involve investment in work, obsessive passion is different from workaholism, where employees are compelled to participate in work activities due to uncontrollable inner pressures and persistence regardless of the consequences without internalizing work as part of their identity and genuinely valued the work itself (Taris and de Jonge, 2024). In sum, we argue that JI will facilitate the development of obsessive passion for the following reasons.

First, unlike other adverse work contexts, JI constitutes a distinct form of resource threat that portends the loss of both employment itself and secondary gains attached to it (Long et al., 2022). According to the resource investment principle of COR theory, employees who perceive JI are likely to exert great effort to prevent loss from becoming reality. This leads to work activity being disproportionately over-occupied employees’ identity, whereby they are overly immersed in their work. In other words, JI may trigger an uncontrollable urge, thereby leading employees internalizing their work in a controlled manner (Vallerand and Houlfort, 2019). Under such conditions, employees engage in work activities not because of intrinsic enjoyment, but because they are tied to instrumental outcomes that matter to the individual.

Second, the threatening context of JI can heighten perceptions of a competitive climate, shifting employees’ focus toward social comparison and the preservation of their relative standing. In this environment, employee outcomes will be evaluated in terms of social comparison rather than self-referenced standards. Thus, this pressure will amplify employee’s tendency to tie self-worth to their job, thereby giving rise to an ongoing quest to preserve their contingent self-esteem and driving compulsive work involvement (Wang et al., 2023). In other words, job-insecure employee may invest excessive effort and resources in work to maintain or improve their performance and relative standing, or at least to meet organizational expectations. Prior studies have shown that low self-control capabilities and self-esteem deficits are predictors of obsessive passion (Breu and Yasseri, 2023). Similarly, research based on job preservation theory suggests that employees with higher JI tend to adopt protective behaviors, such as impression management, avoid-performance goal orientation and facades of conformity, as means to demonstrate their worth and avoid marginalization (Hewlin et al., 2016; Long et al., 2022; Ma et al., 2023). Therefore, we argue that higher levels of JI foster controlled internalization of work, thereby promoting obsessive passion.

*Hypothesis* 1: Job insecurity is positively related to obsessive passion.

In contrast, harmonious passion arises from an autonomous internalization process, wherein employees voluntarily integrate their work into their identity driven by the congruence between job characteristics and their self without any contingencies attached (Vallerand et al., 2003). Drawing on DMP, cultivating and maintaining harmonious passion requires sustained investment of cognitive, emotional, and behavioral resources and positive environment (Vallerand, 2015; Shen et al., 2023; Wu and Zi, 2024). Accordingly, when employees perceive that JI threatens their access to valued resources, they may reduce emotional attachment and cognitive involvement with their work, either consciously or unconsciously, to preserve remaining resources and avoid a downward spiral of resource loss.

In addition, the autonomous internalization of work activity depends on the alignment between their work and employee’s self-expression and satisfaction of basic psychological needs (Zhang et al., 2025). Based on logic above, a higher sense of JI makes the attainment of instrumental goals an active driving factor for employees’ participation in work activities, driven more by the need to secure attached resources than by the intrinsic value of the task. It may disrupt employees’ intrinsic evaluation of their work, weaken its identity-based meaning and thereby undermine the psychological foundation of harmonious passion. Meanwhile, previous studies have also indicated that JI could undermine basic psychological needs (Vander Elst et al., 2012; Vander Elst et al., 2014). In sum, this study posits that JI impairs employees’ autonomous internalization of work activities, and consequently, thus diminishing harmonious passion.

*Hypothesis* 2: Job insecurity is negatively related to harmonious passion.

#### 2.2.2 Mediating role of dualistic passion

##### 2.2.2.1 Mediating role of obsessive passion

According to the DMP, obsessively passionate individuals are compelled to engage in work activities in a manner that is rigid and uncontrollable. For a long time, most theory and empirical research consistently indicates that obsessive passion may lead to intense conflict between work and other life domains (Dubreuil et al., 2014; Vallerand et al., 2014; Kong and Ho, 2018; Rahman et al., 2022). Nonetheless, its motivational force can also drive employee to pursue contingent self-esteem, organizational acceptance, and valued rewards, which can facilitate resource acquisition in the face of adversity (Astakhova, 2015; Astakhova and Porter, 2015). As such, obsessive passion may be a critical motivational force that enables employees to persist and not withdraw when facing difficulties or threats (Vallerand, 2015). This is particularly salient in contexts involving potential job loss, where obsessively passionate individuals safeguard their employment and protect the contingencies associated with it. According to the principles of COR theory, *“when resource loss circumstances are high, resource gains become more important”* (Hobfoll et al., 2018). That is, resource investment functions as a primary mechanism through which individuals mitigate the risk of potential resource loss. Furthermore, the strength model of self-control also offers substantiation for the hypothesis that self-control failures may be attributable to the partial depletion of resources. Motivational salience, such as that derived from the importance of work, can activate employee to utilize the remaining resources, and then persist in work behaviors despite the presence of stress (Baumeister and Vohs, 2016).

As mentioned above, dissatisfaction with the status quo and the discrepancy between current conditions and desired outcomes makes “proactivity matters” for employees. Therefore, we argue that in the context of JI, task proactivity, which entails direct efforts to optimize how tasks are performed, can function as a particularly effective coping strategy, and this relationship is mediated by obsessive passion.

First, task proactivity is an efficient way for employees to enhance their competence in the short term (Strauss and Parker, 2018). Obsessive passion drives employee to focus narrowly on task goals with the sole aim of achieving performance outcomes. As such, employees with obsessive passion are more likely to exhibit a “want to” or “aspire to” form of motivation for task proactivity, fulfilling their goals or aspirations of improving short-term performance. This may help them build instrumental skill-based resources to buffer the possibility of real unemployment in the future. Meanwhile, it sends a signal of their competence and value to the manager, thereby strengthening their control over current work resources (Whiting et al., 2012; Choi and Moon, 2017; Park et al., 2022).

Second, task proactivity allows employees to protect their self-worth through instrumental self-representation. Because the core purpose of task proactivity lies in the improvement and optimization of work processes, it is typically interpreted by managers as responsibility and organizationally goal orientation rather than impression management or turnover intention (Ma et al., 2023). Consequently, such behaviors are more likely to enhance supervisor performance evaluations and minimize interpersonal risk. Indeed, numerous studies have shown that obsessive passion can promote job engagement (Slemp et al., 2021; Teng et al., 2021; Landay et al., 2022). Meta-analytic evidence suggests that obsessive passion positively relates to self-efficacy and organizational citizenship behaviors (Pollack et al., 2020). Furthermore, research on JI has shown that employees are motivated to protect their relative performance standing in the organization under defensive goal orientations (Tu et al., 2024). Therefore, we propose that obsessive passion mediates the relationship between JI and task proactivity.

*Hypothesis* 3a: Obsessive passion is positively related to task proactivity.

*Hypothesis* 3b: Obsessive passion mediates the relationship between job insecurity and task proactivity. Specifically, job insecurity has a positive indirect effect on task proactivity through obsessive passion.

##### 2.2.2.2 Mediating role of harmonious passion

Although employees may proactively respond to JI with obsessive passion, COR theory also guides us to think about the cost of JI. With high levels of harmonious passion, employees engage in work with flexibility, focus, and freeness, owing to a high degree of alignment between the work and their pre-existing values and identity (Vallerand and Houlfort, 2019). Such employees voluntarily invest substantial time and energy in self-defining work activities. Positive emotional experiences, including optimism, happiness, and confidence, can be cultivated merely by doing the work itself (Dubreuil et al., 2014; Appu and Sia, 2017; Tolentino et al., 2022). Furthermore, they are more prone to pursue self-endorsed goals, experience strong feelings of volition, and exhibit heightened self-determination. This, in turn, facilitates task focus and goal attainment under the guidance of the “true self.” Consequently, we argue that harmonious passion is expected to positively predict proactivity and serve as a negative mediating mechanism through which JI influences task proactivity.

Proactive behavior, a resource-intensive one, requires continuous investment of higher-order cognitive and emotional functions (Strauss and Parker, 2018). Meanwhile, from the outcome-oriented perspective, proactivity also empowers employees to obtain and accumulate valuable competence as well as to withstand pressure, thereby facilitating sustainable functioning (Cangiano et al., 2019; Matsuo, 2025). Based on COR’s resource investment logic, individuals are motivated to pursue new, valuable resources to achieve meaningful goals (Hobfoll et al., 2018). Those with sufficient resources are more likely to exhibit the cognitive flexibility and regulatory capacity needed to invest. In contrast, those with fewer resources may not be sufficiently motivated. In this sense, harmonious passion serves as a crucial and resilient personal resource, providing abundant and sustainable motivation (Zhang et al., 2025). The intrinsic enjoyment derived from the activity enables individuals to proactively improve work methods. Employees with high harmonious passion integrate work into an important yet balanced part of their identity and view task accomplishment as a means of expressing their authentic self. Therefore, we propose that harmonious passion mediates the relationship between JI and task proactivity.

*Hypothesis* 4a: Harmonious passion is positively related to task proactivity.

*Hypothesis* 4b: Harmonious passion mediates the relationship between job insecurity and task proactivity. Specifically, job insecurity has a negative indirect effect on task proactivity through harmonious passion.

### 2.3 Moderating role of performance climate

COR theory suggests that the perceived value and accessibility of critical resources shape employees’ resource management strategies (Hagger, 2015; Ma et al., 2023). In fact, only when resources are perceived as both valuable and accessible are individuals motivated to protect and invest in them. As noted earlier, the salience of resource value attached to work will be amplified by JI. Therefore, employees’ decisions to invest in resource-intensive strategies, such as task proactivity, depend heavily on their perceived accessibility of those resources.

Perceived motivational climate refer to “employees’ perceptions of the extant criteria of success and failure, which is emphasized through the policies, practices, and procedures of the work environment” (Nerstad et al., 2013a). This study mainly focuses on the performance climate dimension, which encourages enhancement of normative ability, social comparison with colleagues, intra-team competition and emphasizes the achievement of goals relative to others (Nerstad et al., 2013b). Such climates may compel employees to act proactively while also shaping how they interpretation of success, failure, and organizational expectations. Thus, this study hypothesizes that while the performance climate may reinforce the degree of resource threat, it is also an important factor affecting the employee’s judgment of whether the task proactivity will ultimately lead to resource acquisition.

Specifically, we first propose that performance climate may be a significant moderator that can strengthen the positive effect of JI on obsessive passion. On the one hand, in a higher performance climate, manager focus on relative goal attainment and performance outcomes. This makes it necessary for employees to continuously pay attention to their own performance and closely monitor their own and others’ work results to assess their relative position within their team or organization (Cerne et al., 2014). This constant social monitoring encourages employees to devote a disproportionate amount of time and effort to maintaining their relative advantage. Accordingly, under JI, the performance climate is more likely to drive employees to over-engage at work in order to secure organizational acceptance and position stability. On the other hand, from the perspective of norm theory, performance climate can also be regarded as a specific manifestation of a injunctive norm in the organization, that is, the norm of how employees perceive the organization expects them to behave (Wenzel and Woodyatt, 2025). When the performance climate is high, managers will send a series of normative signals to employees through goal setting, result-oriented feedback mechanisms, and performance reward and punishment systems: high performance is expected and rewarded behavior. Thus, in order to gain group acceptance and avoid the risk of being perceived as low-value, employees will tend to consciously follow performance norms. In other words, when employees perceive JI, this normative pressure compels them to respond to uncertainty with higher levels of work engagement, thus exacerbating the formation of obsessive passion (Miller and Prentice, 2016).

In addition, this study assume that performance climate may also enhance the negative effects of JI on harmonious passion. On the one hand, the externally focused goal structure of performance climate encourages employees to view work as a means to gain acceptance and rewards (Nerstad et al., 2013b). This leads employees to be more inclined to regard work activities as instrumental behavior which is necessary to achieve external goals (such as performance evaluation and promotion and reward). Thereby, it increases employee more instrumental engagement and undermines autonomous identification with the work (Wu and Zi, 2024). Moreover, performance climate often accompanied with result-oriented feedback mechanism and visible performance reward and punishment system. It further reduces employees’ autonomy in choosing how to work, and weakens their sense of control and meaning in work activities, thus inhibiting their autonomous internalization process. Because the continuous requirement of social comparison with others and the highly visible evaluation system will decrease employees’ perceived autonomy, which will lead to cognitive self-threat, thus aggravating the cognitive basis for destroying harmonious passion.

Taken together, in high performance climates, JI is more likely to induce obsessive passion driven by social norms and competitive pressure, thus promoting short-term task proactivity. On the contrary, that climate also will significantly inhibit employee’s harmonious passion by increasing instrumental motives and depleting psychological resources, thus weakening their capacity to sustain proactive engagement. Therefore, we propose the following Hypotheses 5 and 6:

*Hypothesis* 5a: Performance climate moderates the positive relationship between job insecurity and obsessive passion. Specifically, the relationship is stronger when performance climate is high than when it is low.

*Hypothesis* 5b: Performance climate moderates the positive indirect relationship between job insecurity and task proactivity via obsessive passion. Specifically, the indirect effect is stronger when performance climate is high than when it is low.

*Hypothesis* 6a: Performance climate moderates the negative relationship between job insecurity and harmonious passion. Specifically, the relationship is stronger when performance climate is high than when it is low.

*Hypothesis* 6b: Performance climate moderates the negative indirect relationship between job insecurity and task proactivity via harmonious passion. Specifically, the indirect effect is stronger when performance climate is high than when it is low.

**Figure 1:**
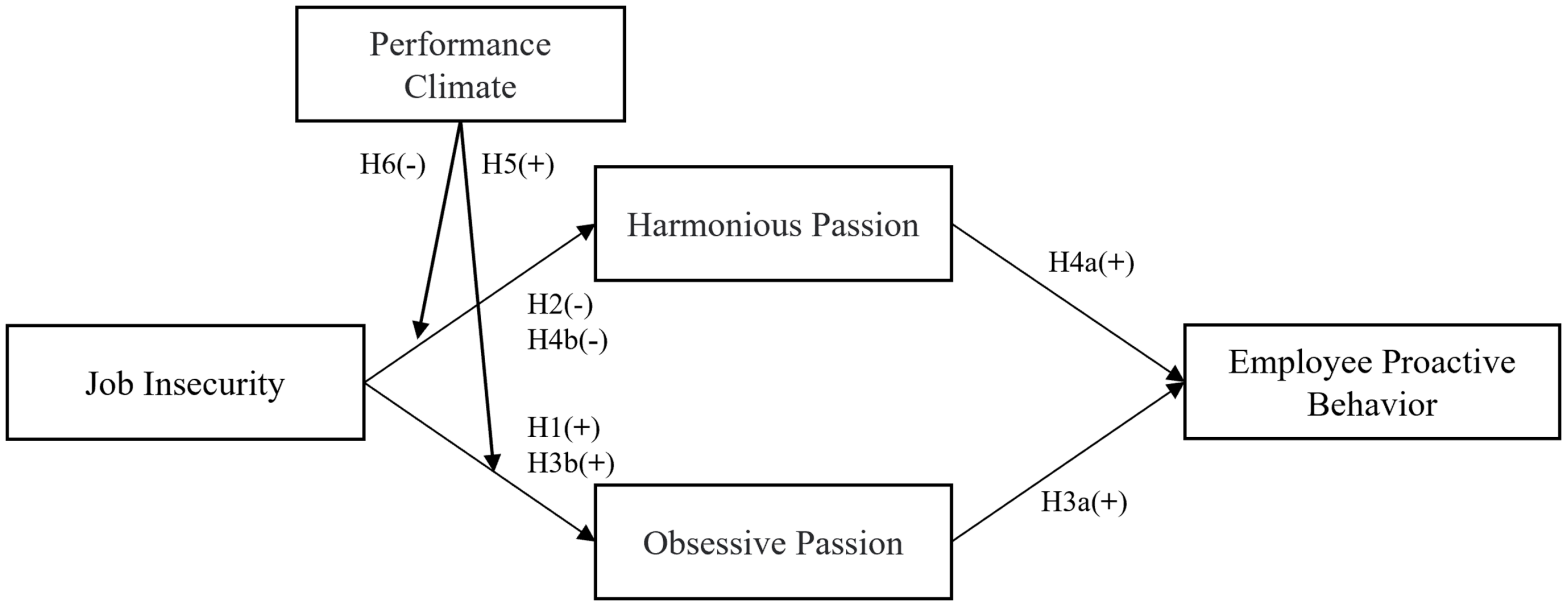
Research model.

In accordance with the discussion above, the research model is shown in Figure 1.

## 3 Method

### 3.1 Participants and procedures

The data for this study were obtained through a two-wave survey conducted among employees from various companies and industries in central and eastern China. Participants primarily consisted of business personnel, administrative staff, engineers and service personnel from internet, financial, business services, and manufacturing enterprises. The collection process was carried out as follows. Prior to the formal survey, the researchers leveraged research projects and social networks to establish contact with participating organizations and obtained their support through full communication. With the assistance of the managers, we randomly invited employees to participate in the study voluntarily, and asked them to fill it out carefully. To mitigate potential common method bias, a time-lagged data collection design was employed. In addition, participants were assured of the academic purpose and that all their responses would be kept strictly confidential.

All questionnaires were distributed during workdays. In the first wave of the survey, data were collected on employees’ demographic characteristics, JI, employee perceived performance climate. In the second wave, data were collected on harmonious passion, obsessive passion and task proactivity, and identifiers used to match responses across waves. A total of 500 questionnaires were distributed in the first wave, with 458 valid questionnaires retained after excluding those with excessive missing data or discernible patterns. In the second wave (2 week later), 412 questionnaires were returned, of which 384 were successfully matched with the first-wave. Among the final sample, 45.8% were male and 54.2% were female. The average age of participants was 34.73 years (*SD* = 6.737), and 60.2% held a bachelor’s degree as their highest level of education. The average organizational tenure was 6.036 years (*SD* = 5.096).

### 3.2 Measures

The scales used in this study are all well-validated and drawn from highly cited literature. In order to ensure the validity of the scale, the English questionnaire was translated into Chinese in strict accordance with the “translation-back translation” procedure during the translation process, and the final questionnaire was formed after adjustment and revision according to the opinions of two doctoral students of relevant majors. Unless otherwise specified, the variables were measured using 5-point Likert scales (1–5 scale ranges from “strongly disagree” to “strongly agree”). The items for all scales is provided in the Appendix.

#### 3.2.1 Job insecurity

We measured job insecurity using the 3-item scale developed by Hellgren and Sverke (2003). A sample item was “I am worried about having to leave my job before I would like to.” Cronbach’s *α* for the scales was 0.842.

#### 3.2.2 Proactive behavior

A three-item scale developed by Griffin et al. (2007) was used to measure task proactivity. Participants were asked to respond how frequently they engaged in the following behavior over the last several weeks. The sample items are “initiated better ways of doing your core tasks” (1 = “much less than usual,”5 = “much more than usual”). Cronbach’s α for the scales was 0.857.

#### 3.2.3 Harmonious passion and obsessive passion

Both harmonious and obsessive passion were measured using the 14-item scale developed by Vallerand et al. (2003), with slight modifications to better reflect the work context in this study. Sample items are “This job allows me to live a variety of experiences” and “The urge is so strong. I cannot help myself from doing my job.” Cronbach’s α for the scales of harmonious passion was 0.896, and for the scales of obsessive passion was 0.922.

#### 3.2.4 Performance climate

We measured performance climate using the adapted 7-item scale developed by Nerstad et al. (2013a). A Sample item includes “In my work group, it is important to achieve better than others.” Cronbach’s α for the scales was 0.936.

#### 3.2.5 Control variables

Following prior researches, we controlled for employee gender (0 = male, 1 = female), age, education (1 = junior college and below, 2 = undergraduate college, 3 = postgraduate and above) and tenure four demographic variables to avoid possible effects.

## 4 Results

### 4.1 Descriptive statistics analysis

Descriptive statistics for all variables are present in Table 1. The results showed that there was no significant relationship between JI and task proactivity (*r* = −0.095, *p* > 0.05). In addition, JI was positively related obsessive passion (*r* = 0.481, *p* < 0.01) and negatively related to harmonious passion (*r* = −0.261, *p* < 0.01) respectively. The results of the correlation analysis were basically consistent with the theoretical expectations.

**Table 1:** Correlations, means, standard deviations, AVEs and C. R. among variables.

Variables	Mean	SD	1	2	3	4	5	6	7	8	9	CR
1. Gender	0.460	0.499										
2. Age	34.730	6.737	−0.096									
3. Education	2.260	0.792	−0.025	0.140^**^								
4. Tenure	6.036	5.096	−0.111^*^	0.613^**^	0.202^**^							
5. JI	2.556	0.926	0.006	−0.011	0.099	−0.042	0.801					0.843
6. HP	3.815	0.655	0.028	0.069	−0.058	0.082	−0.261^**^	0.744				0.897
7. OP	3.069	0.912	0.062	0.021	0.120^*^	0.029	0.481^**^	0.031	0.797			0.924
8. TP	3.773	0.747	0.042	−0.037	−0.048	−0.062	−0.095	0.353^**^	0.320^**^	0.817		0.858
9. PC	3.115	0.907	−0.003	0.026	−0.016	0.034	−0.012	0.033	0.347^**^	0.222^**^	0.824	0.937

JI, job insecurity; TP, task proactivity; OP, obsessive passion; HP, harmonious passion; PC, performance climate. The square root of AVE is in parentheses on the diagonal. **p < 0.01; *p < 0.05.

### 4.2 Confirmatory factor analysis

In order to analyze the distinctiveness of the study variables prior to hypothesis testing, we conducted a confirmatory factor an analysis (CFA) using AMOS 20.0 on the five key constructs: JI, harmonious passion, obsessive passion, task proactivity, and performance climate. As shown in Table 2, the hypothesized five-factor model was demonstrated acceptable (χ^2^/ *df* = 2.430, CFI = 0.934, TLI = 0.920, IFI = 0.934, RMSEA = 0.058). We further compared this model to alternative four-factor models. Results indicated that the hypothesized five-factor model fit significantly better than all competing four-factor models (see Table 2), supporting the discriminant validity of the constructs. In addition, CR and AVE were calculated for each construct based on the hypothesized model. As shown in Table 1, all CR and AVE values exceeded recommended thresholds, providing further support for the constructs’ discriminant and structural validity.

**Table 2:** Result of confirmatory factor analysis (CFA).

Model	χ^2^	df	χ^2^/df	CFI	TLI	IFI	RMSEA
Five-factor model	763.046	314	2.430	0.934	0.920	0.934	0.058
Four-factor model (combing JI and TP)	1389.524	318	4.370	0.842	0.812	0.843	0.089
Four-factor model (combing HP and OP)	2153.557	318	6.772	0.729	0.678	0.732	0.117
Four-factor model (combing PC and OP)	2436.910	318	7.663	0.687	0.628	0.690	0.125
Four-factor model (combing JI and OP)	1169.743	318	3.678	0.874	0.851	0.875	0.079
Three-factor model (combing JI, OP and HP)	2540.212	321	7.913	0.673	0.614	0.675	0.128
Two-factor model (combing JI, OP, HP and TP)	3009.630	323	9.318	0.604	0.536	0.607	0.140
Single-factor model (combing all variables)	4748.015	324	14.654	0.347	0.239	0.353	0.179

Since all variables were self-reported by employees, we assessed the potential for common method bias. Harman’s single-factor test shows that the first unrotated factor accounted for only 26.832% of the total variance, well below the 40% threshold. Therefore, it could be considered that our findings are unlikely to be significantly affected by common method variance.

### 4.3 Hypothesis test

Multiple linear regression was firstly employed to conduct a basic test. Subsequently, SEM (based on Mplus 8.3; Muthén and Muthén, 2017) was used to test the mediating effect and the moderated mediating effect. Hypothesis 1 and 2 predicted that JI is positively related to obsessive passion and negatively related to harmonious passion. Model 1 and Model 3 in Table 3 indicated that the effect of JI on obsessive passion and harmonious passion was significant (*β* = 0.476, *p <* 0.001; *β* = −0.253, *p <* 0.001). Therefore, these results supported Hypothesis 1 and 2.

**Table 3:** Hierarchical regression results of mediated and moderated role.

Variables	Obsessive passion		Harmonious passion		Task proactivity		
	Model 1	Model 2	Model 3	Model 4	Model 5	Model 6	Model 7
Gender	0.066	0.061	0.039	0.040	0.036	−0.003	−0.002
Age	−0.005	−0.015	0.040	0.041	0.007	−0.003	−0.005
Education	0.066	0.072	−0.050	−0.049	−0.027	−0.041	−0.036
Tenure	0.046	0.044	0.061	0.058	−0.061	−0.098	−0.097
JI	0.476^***^	0.462^***^	−0.253^***^	−0.248^***^	−0.095^†^	−0.227	−0.207^***^
PC		0.323^***^		0.035			0.073
JI × PC		0.147^***^		−0.044			0.034
OP						0.428^***^	0.383^***^
HP						0.286^***^	0.293^***^
F	24.195	33.930	6.374	4.685	1.180	19.643	15.667
R-squire	0.232	0.376	0.066	0.063	0.002	0.254	0.256
ΔR-squire		0.145^***^		0.002		0.252^***^	0.006

All results are presented as standardized coefficients. ***p < 0.001, **p < 0.01, *p < 0.05, †*p* < 0.1.

Hypotheses 3 and 4 predicted that obsessive passion and harmonious passion would be positively and negatively related to task proactivity, respectively, and that they would mediate the relationship between job insecurity and task proactivity. Model 5 and Model 6 in Table 3 suggested that the effect of JI on task proactivity was only marginally significant (*β* = −0.095, *p <* 0.1); the effect of obsessive (harmonious) passion on task proactivity was significant (*β* = 0. 428, *p <* 0.001; *β* = 0.286, *p <* 0.001). Furthermore, the bootstrapping method proposed by Edwards and Lambert (2007) (bootstrap = 2000) was used to calculate the mediating effect of obsessive passion and harmonious passion. The results showed that the indirect effect of JI on task proactivity through obsessive passion was 0.230, and the 95% confidence interval did not include 0 (95% CI = [0.154, 0.325]); the indirect effect of JI on task proactivity through harmonious passion was −0.072, and the 95% confidence interval did not include 0 (95% CI = [−0.119, −0.039]). Therefore, Hypothesis 3 and 4 were support.

Hypothesis 5 and 6 suggested that performance climate would moderate the effect of JI on obsessive passion and harmonious passion as well as the subsequent mediating effect. Model 2, Model 4 and Model 7 in Table 3 suggested that the interactive effect of JI and performance climate on obsessive passion was significant (*β* = 0.147, *p* < 0.001); however, interactive effect on harmonious passion was not significant (*β* = −0.044, *p* > 0.05). We then used Mplus 8.3 to test hypothesis 5b and 6b. The results in Table 4 indicated that when performance climate was higher (+ 1 SD), the indirect effect of obsessive passion was stronger (Estimate = 0.279, SE = 0.059, 95% CI [0.176, 0.406]) than performance climate was lower (−1 SD; Estimate = 0.136, SE = 0.046, 95% CI [0.061, 0.241]), the moderating effect difference was 0.144 (SE = 0.051, 95% CI [0.056, 0.259]). On the contrary, the moderating effect of the performance climate on the mediating role of harmonious passion was not significant, the moderating effect difference was −0.012 (SE = 0.038, 95% CI [−0.082, 0.069]). Thus, hypothesis 5 was supported, hypothesis 6 was not supported.

**Table 4:** Results of the conditional indirect effects.

Effect type		Moderator level	Estimate	SE	*p*-value	95%CI
Indirect effect via obsessive passion		Low (M-1SD)	0.136	0.046	0.003	[0.061, 0.241]
		High (M-1SD)	0.279	0.059	<0.001	[0.176, 0.406]
Indirect effect via harmonious passion		Low (M-1SD)	−0.065	0.032	0.041	[−0.143, −0.013]
		High (M-1SD)	−0.078	0.024	0.001	[−0.138, −0.038]
Moderating Effect Difference	Obsessive passion path	High-low level difference	0.144	0.051	0.005	[0.056, 0.259]
	Harmonious passion path	High-low level difference	−0.012	0.038	0.753	[−0.082, 0.069]

Moderated variable is performance climate.

**Figure 2:**
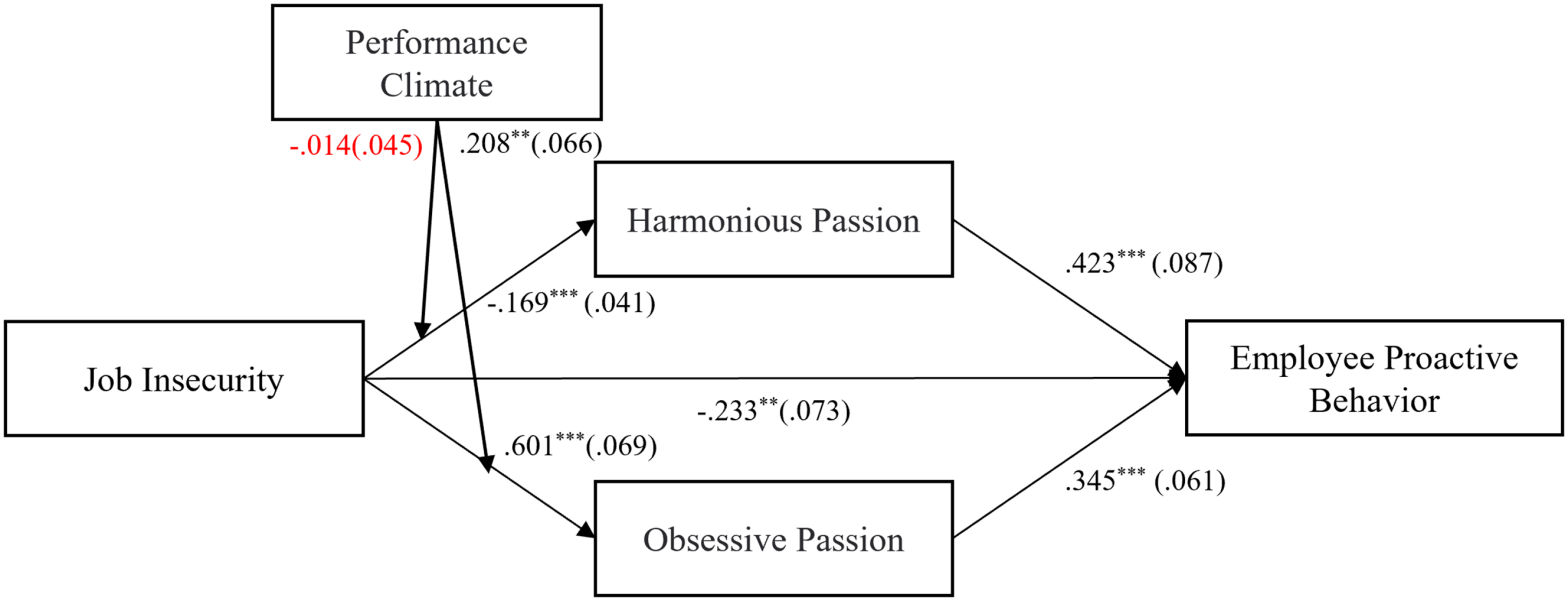
Unstandardized estimates of the moderated indirect effect model. ***p < 0.001, **p < 0.01.

**Figure 3:**
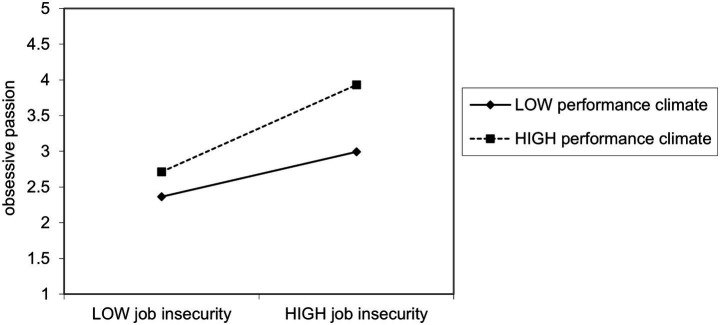
The moderating role of PC on the relationship between JI and OP.

In summary, the results of the SEM test are shown in Figure 2, and Figure 3 shows the simple slope regression lines of JI on obsessive passion for lower (+1SD) and higher (+1SD) levels of performance climate based on the result of hierarchical regression.

## 5 Discussion

Thus far, the literature indicates that the relationship between JI and employee’s proactive behavior is not consistent (Shoss et al., 2022; Klug et al., 2024). Therefore, drawing on COR theory and DMP, we developed and examined a dual-path model to explore why and how employees with JI may engage in proactive behavior to cope with threats. Our results demonstrate that there is only marginal negative significant relationship between JI and task proactivity. However, JI positively influenced task proactivity through obsessive passion, while negatively influencing it via harmonious passion. Furthermore, the positive relationship between JI and obsessive passion was stronger under high levels of performance climate, thereby associated with higher task proactivity. In contrast, we found no evidence that performance climate moderated the relationship between JI and harmonious passion.

The lack of a moderating effect of performance climate on the harmonious passion path may stem from its formation mechanism. DMP suggests that a supportive environment serves as a crucial antecedent for employees’ autonomous internalization of work. Therefore, regardless of whether performance climate is high or low, it may fail to provide a conducive environment for nurturing employees’ harmonious passion. Consistent with our findings, Zhang Q, et al. (2022) also reveal that the correlation between performance climate and harmonious passion is insignificant. On the other hand, under high JI conditions, the detrimental effect of JI on the psychological foundations of harmonious passion is likely more direct and universal. Consequently, a high performance climate may not necessarily amplify this negative impact.

### 5.1 Theoretical implications

This study makes several theoretical contributions to the literature. First, this study contributes to explaining the paradoxical findings regarding the relation between employee’s JI and proactive behavior. While previous studies have emphasized the forces of propelling and restraining separately that job insecure employee may be faced, few studies have integrated these competing mechanisms within a single framework. According to COR theory, we have discussed the different mechanisms of obsessive passion and harmonious passion on the correlation between JI and employees’ task proactivity: resource depletion and resource investment mechanism. Consistent with previous findings, the findings show that JI undermines the psychological foundation of harmonious passion which in turn inhibits proactive behavior through a resource depletion mechanism. Whereas, JI also heightens employees’ instrumental valuation of their job and the need to maintain contingent self-esteem, thereby stimulating obsessive passion and triggering adaptive, effortful responses as a form of resource investment. By integrating these dual pathways into one model, our findings explain the underlying suppression effect in the relationship between JI and task proactivity, thus answering recent calls for research into the paradoxical consequences of JI.

Second, this study extends the theoretical boundaries of the DMP in adverse organizational contexts, shedding light on the potential constructive role of obsessive passion under high-pressure situations. Traditional research has predominantly associated obsessive passion with negative outcomes such as rigid behavior and psychological exhaustion, implying that as a controlled form of motivation, it is inherently detrimental to individual adaptation (Kong and Ho, 2018; Pollack et al., 2020; Landay et al., 2022). However, the findings of this study reveal that in the context of JI, obsessive passion may not only stimulate stronger motivations for resource preservation and self-presentation but also promote engagement in goal-oriented proactive behaviors to maintain a sense of control over the work environment. This discovery challenges conventional perspectives by highlighting the context-dependent functionality of motivation. It indicates that, in specific high-stress situations, obsessive passion may act as a functional coping mechanism in the short term, driving individuals to take positive actions to mitigate identity threats and the risk of resource loss. However, it is important to note that it would be unwise to interpret obsessive passion as an unqualified benefit. A substantial body of literature suggests obsessive passion may indeed have negative effects on well-being and other employee outcomes in the long run. Therefore, we posit that while obsessive passion can channel employee’s resources into proactivity in the face of threat, if external pressures continue without resolution, the aforementioned negative consequences will inevitably manifest. This study advocates for a more dialectical approach to understanding the functional mechanisms of obsessive passion in future research, particularly within unstable employment environments.

Third, this study extends the COR theory by identifying the boundary conditions under which resource investment becomes a viable coping strategy in the face of JI. While COR theory has traditionally emphasized the importance of protecting against resource loss, recent work calls for more attention to the contextual factors that shape individuals’ willingness to invest resources when threatened. We argue and demonstrate that a high-performance climate, characterized by normative performance expectations, and competitive comparison, can serve as a salient injunctive norm that clarifies the instrumental value of proactive behavior. In doing so, it strengthens the perceived utility of resource investment and encourages employees to engage in task-focused proactivity, even under threatening conditions. This perspective advances COR theory by highlighting not only the motivational significance of resource gain but also the work contextual signals that reinforce resource investment decisions.

### 5.2 Practical implications

Our findings offer actionable insights for mitigating the adverse effects of JI. In light of the current global economic downturn and the intensifying domestic and international competitive pressures faced by organizations, JI often originates from external forces beyond the organization’s control. In other words, organizations can alleviate, but not entirely eliminate, the negative consequences of JI. Our results support this perspective: employees adopt different resource-based strategies to cope with JI depending on their levels of work passion. Accordingly, one practical implication of this study is that organizations should implement measures to channel employees’ energy toward more adaptive outcomes. Specifically, managers can facilitate this process by providing clear performance structures and timely recognition of work achievements, thereby enhancing the instrumental value of proactive behavior. However, it is critical for organizations to monitor employees for signs of overcommitment and excessive workload, as these may lead to long-term costs.

Furthermore, our findings underscore the instrumental role of performance climate in shaping employees’ cognitive evaluations of whether proactive behavior constitutes a worthwhile investment under JI. A performance climate characterized by clearly defined success criteria and competitive norms can strengthen the perceived link between positive efforts and valuable outcomes. However, managers should also be extremely cautious about this strategy. An overemphasis on competition and normative performance can easily result in unintended consequences, such as emotional exhaustion, or unethical behaviors aimed at merely outperforming others. Thus, managers’ goal should not be solely to create a competitive environment, but to foster healthy intra-team competition and clarify the connection between tasks and results. These signals not only help align employees’ resource investments with organizational expectations but also buffer, to some extent, the ambiguity and anxiety associated with JI. At the same time, it is essential for managers to ensure that competition does not devolve into destructive rivalry, while also monitoring employee well-being.

### 5.3 Limitations and directions for future research

Despite some theoretical contributions this study provides, there are several limitations that should be noted. First, all of our data were collected through self-reported questionnaires, which raises the concern of common method bias. Although we adopted several ex ante procedural control and statistical tests to mitigate and assess this issue, and the results suggest that our findings are unlikely to be seriously affected by common method variance, we cannot completely rule it out. Moreover, the cross-sectional nature of the data prevents us from making strong causal inferences about the relationships among the variables. Furthermore, the two-wave survey within two-week interval might be too short to capture fully changes of work passion and proactivity. In addition, given the current social context, the sources of employees’ JI are likely to be multifaceted, and the formation of coping strategies may involve multiple mechanisms simultaneously. Future research should consider employing qualitative methods to capture employees lived experiences more comprehensively and to uncover the nuanced processes through which individuals respond to JI.

Second, this study primarily focused on the short-term effects of performance climate and obsessive passion in the context of JI. While our findings highlight the adaptive value of controlled or instrumental motivation under conditions of uncertainty and threat, it is critical to note that this may come at a long-term cost, as obsessive passion and performance climate are a known predictors of strain and burnout. Future research should employ longitudinal designs to track the evolution of these effects over time. It is crucial to investigate when and for whom the short-term benefits of obsessive passion might be outweighed by its long-term harms.

Lastly, our data were collected in China, a cultural context characterized by collectivism and high-power distance. In such a context, the social comparison and competitive pressures inherent in a performance climate might be particularly potent, as they align with norms of fulfilling collective expectations and respecting hierarchical authority. Consequently, the positive relationship between job insecurity, obsessive passion, and task proactivity, and its amplification by performance climate, might be especially salient in China. Future research could test our model in diverse cultural settings to establish its cross-cultural validity.

## Data Availability

The raw data supporting the conclusions of this article will be made available by the authors, without undue reservation.

## Appendix

Items measuring job insecurity (Hellgren and Sverke, 2003).

1. I am worried about having to leave my job before I would like to.
2. There is a risk that I will have to leave my present job in the year to come.
3. I feel uneasy about losing my job in the near future.

Items measuring performance climate (Nerstad et al., 2013a).

1. In my work group, it is important to achieve better than others.
2. In my work group, work accomplishments are measured based on comparisons with the accomplishments of coworkers.
3. In my work group, an individual’s accomplishments are compared with those of other colleagues.
4. In my work group, rivalry between employees is encouraged.
5. In my work group, only those employees who achieve the best results are set up as examples.
6. In my work group, internal competition is encouraged to attain the best possible results.
7. In my work group, there exists a competitive rivalry among the employees.

Items measuring harmonious passion (Vallerand et al., 2003).

1. This job allows me to live a variety of experiences.
2. The new things that I discover with this job allow me to appreciate it more.
3. This job allows me to live memorable experiences.
4. This job reflects the qualities I like about myself.
5. This job is in harmony with the other activities in my life.
6. For me it is a passion, that I still manage to control.
7. I am completely taken with this job.

Items measuring obsessive passion (Vallerand et al., 2003).

1. I cannot live without my job.
2. The urge is so strong. I can’t help myself from doing my job.
3. I have difficulty imagining my life without my job.
4. I am emotionally dependent on my job.
5. I have a tough time controlling my need to do my job.
6. I have almost an obsessive feeling for my job.
7. My mood depends on me being able to do my job.

Items measuring task proactivity (Griffin et al., 2007).

Participants were asked to respond how frequently they engaged in the following behavior over the last several weeks.

1. Initiated better ways of doing your core tasks.
2. Come up with ideas to improve the way in which your core tasks are done.
3. Made changes to the way your core tasks are done.
